# Statistical Assessment, Modeling, and Mitigation of Water and Soil Pollution

**DOI:** 10.3390/toxics10050261

**Published:** 2022-05-18

**Authors:** Lucica Barbeş, Alina Bărbulescu

**Affiliations:** 1 Departament of Chemistry and Chemical Engineering, “Ovidius” University of Constanța, 124 Mamaia Bd., 900527 Constanta, Romania; 2 Department of Civil Engineering, Transilvania University of Brașov, 5 Turnului Str., 900152 Brasov, Romania

Nowadays, ambient air pollution levels and trends have become a topic of interest worldwide because primary atmospheric pollutants (APPs) are risk factors for the population and ecosystems. Therefore, monitoring air quality, especially in urban or crowded areas, is essential for controlling pollution and protecting human health [1,2].

This Special Issue covers topics related to statistical analysis and modeling applied to study soil and vegetation pollution, groundwater, and surface water contamination, advancing the existing knowledge.

The leading cause of water pollution is wastewater discharges resulting from domestic and industrial activity. In most cases, these discharges are unavoidable. Their release into the receptors (natural water bodies) must be conditioned so that post-treatment water users are affected as little as possible. In addition, the effluents of many treatment plants are insufficiently depolluted and may contain a wide variety of inorganic, organic, radioactive substances, etc. It is also worth mentioning that wastewater is unsuitable for irrigation even after biochemical treatment.

Anthropogenic soil pollution directly results from the storage and spreading of household waste, liquid and solid residues from industrial activities, and chemical compounds used in agriculture. Insufficiently treated domestic or industrial wastewater is one of the most common sources of soil pollution.

Air pollution can also generate severe surface/groundwater pollution and soil effects in agricultural or forestry areas, accentuating desertification. Other consequences with a substantial impact on the soil also appear in farming or forestry vegetation, including medicinal plants or those with honey potential.

The integrated assessment of the environmental pollution factors is made with the help of statistical methods and mathematical modeling of the data series formed by the contaminants’ concentrations [3].

Out of ten submitted manuscripts, seven papers were selected for inclusion in the present Special Issue after the peer-review process. The complexity of this Special Issue resides in the interpretation of chemical information to correctly indicate the level of pollution associated with the natural and anthropogenic characteristics of the analyzed areas and transpose in mathematical information the experimental results. We believe that the published papers highlight some essential topics related to environmental pollution and the tools for its estimation.

Three articles focus on the examination of soil contamination [4,5,6] and the possible translocation of the pollutants from soil to plants [7], and the rest focus on surface and groundwater quality [8,9,10].

The article by Nugraha et al. [4] presents the results of a study performed in an area with high natural background radiation. The heavy metal content in soil samples from that zone was analyzed, using high-purity germanium (HPGe) gamma spectrometry and flame atomic absorption spectrometry (FAAS). It is shown that the soil in Mamuju contains a high concentration of ^226^Ra and ^232^Th. The concentrations of other metals are in decreasing order: Zn > Pb > Cr > Cu > Ni > Cd, leading to a moderate pollution level of the soil that is estimated based on the ecological risk index (*RI*) and cumulative pollution index (*IPI*). The high correlation between the ^232^Th, ^226^Ra, and Pb found can be explained by the radionucleotides’ decay into different Pb isotopes. Since the Pb concentration exceeded at least four times the allowable limit established by the WHO and US EPA, negative effects on the population’s health may appear.

The articles by Al Taani et al. [5] and Nazal et al. [6] evaluate the soil contamination in three different zones of the Abu Dhabi Emirate, using data collected in two campaigns, mainly from agricultural soils situated in the Liwa area, Madinat Zayed, and along the Abu Dhabi-Al Ain road. The Principal Component Analysis (PCA) and pollution indexes (enrichment factor—*EF*, geo-accumulation index—*Igeo*, pollution load index—*PLI*, ecological risk assessment—*PERI*, the Nemerow pollution index, etc.) are utilized for this purpose.

The results from the first campaign [5] indicate high enrichment of the soils with Cd, Cr, Zn, and Ni with anthropic origin. The *Igeo* found the soil as uncontaminated to moderately contaminated with Ni and moderately contaminated with Cd. Based on the contamination factors, moderate pollution with Ni was noticed, whereas with respect to the *PLI*, the samples were classified as unpolluted to low polluted. The *PERI* indicated a high ecological risk for Cd.

The PCA performed on the 84 series formed by the concentrations of 17 metals collected at each location (of 84) in the second campaign [6] led to extracting four principal components (PCs) for describing the total metal concentrations’ variance. Mn, Cr, Fe, Cu, and Al had the highest contributions to the first four PCs. Grouping the sites using clustering technics resulted in three groups (one with a single element), one of them containing the most polluted soils with Cu, Ni, Zn, and Pb, located mainly along the highways or near petroleum stations.

In article [7], the authors continue the research from [11,12] on the soil pollution and its impact on some melliferous plants (*Sambucus nigra* L., *Hypericum perforatum*, and *Tilia tomentosa*). The flame atomic absorption spectrometry (FAAS) was utilized to determine the macro- and microelement contents in the soils and the flowers, leaves, and stems of the samples of the studied plants collected at three locations in Dobrogea, Romania. Despite statistical tests on the concentrations’ series of different elements collected at the three sampling sites found no difference between the elements’ concentration (as a whole), the values of different pollution indices show various levels of contamination of the sites. The highest accumulation in the soil is that with Cd and Cu, as the most polluted place is situated in a touristic area. Tilia tomentosa acted as a bioaccumulator for almost all studied metals (K, Mg, Na, Fe, Mn, Cu, Zn, and Cd).

The study shows that a complex approach, including statistical tests and the computation of soil pollution indicators, is necessary to correctly evaluate the degree of soil pollution level and the pollutants.

In article [8], the scientists extend the research from [13] on the water pollution of the most important Indian rivers. Based on the series of eight water parameters, they computed three water quality indicators (WQI) based on which the pollution level of the Brahmaputra River (entirely and at each hydrometrical station) is evaluated. Another aspect was to group the hydrological series in a cluster function of the computed WQIs, and to determine the regional and temporal trend of the contamination. Statistical tests failed to reject the hypothesis of a monotonic trend only for the spatial data series, indicating an increasing pollution trend over time.

The article by Mihăilescu et al. [9] proposes the optimization of the Au extraction from industrial solutions using Am-L-GA. This approach is a factorial design in two phases: linear and nonlinear. It was shown that the parameter with the significant contribution to gold extraction is the initial gold concentration in solution, the contact time being a secondary factor. The study indicates that this approach could be successfully employed to identify the main factors involved in the adsorption process and diminish experimental effort, reducing the number of trials and extending the variables’ domains.

De Jesus et al. [8] propose a neural network-particle swarm optimization (NN-PSO) model for evaluating the surface water and groundwater quality by employing the water’s physico-chemical parameters. Comparison with SVM and linear approaches emphasized the best performances of the NN-PSO.
